# Magnetisation transfer, T1 and T2* relaxation in canine menisci of elderly dogs—an *ex vivo* study in stifle joints

**DOI:** 10.3389/fvets.2025.1521684

**Published:** 2025-03-12

**Authors:** Lena Bunzendahl, Amir Moussavi, Martina Bleyer, Stephan Neumann, Susann Boretius

**Affiliations:** ^1^Small Animal Clinic, Institute of Veterinary Medicine, Georg-August University of Göttingen, Göttingen, Germany; ^2^Functional Imaging Laboratory, German Primate Center, Leibniz Institute for Primate Research, Göttingen, Germany; ^3^Fachhochschule Südwestfalen, University of Applied Sciences, Hagen, Germany; ^4^Pathology Unit, German Primate Center, Leibniz Institute for Primate Research, Göttingen, Germany

**Keywords:** multi-parameter MRI, T2* mapping, T1 mapping, magnetisation transfer, canine menisci, osteoarthritis, stifle joint, proteoglycans

## Abstract

Magnetic resonance imaging (MRI) is widely used in human medicine, offering multiple contrast mechanisms to visualise different tissue types. It is also gaining importance in veterinary medicine, including diagnosing joint disorders. The menisci of the stifle joint play a crucial role in the development of osteoarthritis (OA), and multi-parameter MRI of the menisci may aid in early OA diagnosis, potentially improving therapeutic outcomes. In a previous *ex vivo* study, we measured T2 relaxation times in menisci of elderly dogs with mild histological signs of degeneration but no clinical symptoms of lameness. As no significant changes in T2 relaxation times were observed in relation to histological scores, the present study extends this investigation by exploring more advanced MR parameters—including T1 relaxation time, T2* relaxation time, magnetisation transfer ratio (MTR), and magnetisation transfer saturation (MTsat)—to assess their potential for detecting early microstructural changes in the menisci. While T2* relaxation times and MTR showed no significant variation across histological scores, MTsat values increased with higher proteoglycan staining. In contrast, the apparent T1 relaxation time (T1app) was lower in menisci with elevated proteoglycan scores and increased with higher cellularity scores. The correlation between MTsat and proteoglycan content suggests that MTsat, along with T1app, could be a promising parameter for characterising the extracellular matrix. However, further research is needed to validate these findings.

## 1 Introduction

Osteoarthritis (OA) of the canine stifle joint is a predominantly degenerative joint disease, especially common in older dogs. OA frequently develops in the context of cranial cruciate ligament disease (1) and is closely associated with damage or degeneration of the menisci. These fibrocartilaginous structures play a critical role in absorbing compressive loads and maintaining joint stability, which helps to protect the articular cartilage from excessive wear (2–4). As OA progresses, it leads to structural changes in all joint components, including the medial and lateral menisci, making meniscal degeneration a key factor in OA onset and progression.

Magnetic resonance imaging (MRI) is the gold standard for diagnosing knee disorders in humans, offering high-resolution, non-invasive imaging of soft tissues and bone structures. It is routinely employed to detect meniscal tears, ligament injuries, and cartilage degeneration (5–12). In veterinary medicine, although traditionally less common, the use of MRI is steadily increasing (13, 14). In addition to identifying macroscopic injuries (10, 11, 15), MRI is gaining importance in characterising tissue microstructures. Recent advances in quantitative and semi-quantitative techniques have proven particularly useful for assessing microscopic changes in humans (9, 16–18) and animals (1, 19–21).

The menisci are composed of fibrochondrocytes embedded in an extracellular matrix primarily consisting of water, collagen, and proteoglycans (22–25). The collagen fibres, primarily type I with smaller amounts of type II and III, are arranged in radial and circumferential patterns, allowing the menisci to resist multidirectional stresses. Proteoglycans, mainly aggrecan, make up 1–2% of the meniscal dry weight and contain glycosaminoglycan (GAG) chains such as chondroitin sulphate and keratan sulphate (22).

In osteoarthritic menisci, alterations in the extracellular matrix commonly lead to increased water content and mobility, which in turn influences the T2 relaxation time. Elevated T2 relaxation times have been documented in human meniscus degeneration (9, 10). In a previous *ex vivo* study, we measured T2 relaxation times in menisci from elderly dogs with mild histological degeneration but without clinical signs of lameness. The results showed no significant changes in T2 relaxation times with higher histological scores, suggesting that these early degenerative changes did not markedly affect the T2 values (26). We have now expanded our investigation to include T1 and T2* relaxation times, as well as magnetisation transfer (MT) techniques and the derived parameters, including magnetisation transfer ratio (MTR) and magnetisation transfer saturation (MTsat).

These advanced MR parameters are not yet part of routine clinical diagnostics in human or veterinary medicine, partly due to longer acquisition times and the complexity of data interpretation (27, 28). However, these techniques are gaining significance in medical imaging (27), and *post mortem* studies may help to identify promising candidates for future clinical applications. In the following, we will briefly address the potential advantages and added value of these techniques for the microtissue characterization of the menisci.

Given the observed increase in water content during meniscal degeneration, T2* relaxation times may behave similarly to T2 relaxation times. Nebelung et al. reported higher T2* relaxation values correlating with increasing histological scores in humans, though primarily at the extreme ends of the spectrum (29). Furthermore, Lee et al. found a positive correlation between contact strain and T2* relaxation times in the articular cartilage of cattle (6). Although T2 and T2* are related, T2* is more sensitive to magnetic field inhomogeneities, potentially providing additional insights into subtle tissue changes such as variation in fiber density and orientation. However, this heightened sensitivity poses challenges for data acquisition and interpretation, limiting its current clinical use.

T1 relaxation time, another parameter sensitive to water content, is valuable for assessing cartilage and soft tissues (8, 18, 30, 31), but its application to the meniscus remains largely in the research phase.

MT techniques have been widely applied to indirectly assess macromolecular content in various biological tissues (32–36). These methods exploit the interaction between free water protons and protons bound to macromolecules. By selectively saturating the bound protons, magnetisation is transferred to the free water protons, resulting in a reduction in signal intensity. The MTR quantifies the degree of this signal loss by comparing it to a data set acquired without the saturating radiofrequency pulse. Higher MTR values typically indicate denser macromolecular structures (12). Several studies have measured MTR in articular cartilage and menisci, reporting lower MTR values in tissues with reduced collagen and proteoglycan content (12, 30, 32).

A limitation of MTR is its lack of specificity. Various factors, such as inflammation and oedema, can influence the results. Additionally, MTR is susceptible to B0 (main magnetic field) and B1 (radiofrequency) field inhomogeneities and highly sensitive to acquisition parameters, such as the choice of saturation pulse, which can lead to inconsistent values and makes standardisation across different studies or clinical environments challenging. To address these limitations, quantitative magnetisation transfer (qMT) methods have been developed. These techniques provide more specific and reproducible macromolecular parameters, such as the macromolecular pool size fraction and the exchange rate between free and bound protons. However, the advantages of qMT come at the cost of longer acquisition times and more complex data processing, limiting its availability in clinical practice.

Helms et al. (37) introduced a technique called MTsat that bridges the gap between the simplicity of MTR and the complexity of full qMT imaging. MTsat incorporates corrections for B1 inhomogeneities and T1 relaxation times, providing more accurate data but with shorter acquisition times compared to full qMT. MTsat is increasingly used in research settings, particularly for studying neurological disorders (38–41). To our knowledge, this technique has not yet been applied to examine musculoskeletal structures. We hypothesize that MTsat may be suitable for quantifying the macromolecular content of the meniscus, thereby offering more detailed insights into the remodeling processes of menisci during degeneration.

In this study, we determined the T1 and T2* relaxation times, along with MTR and MTsat, in menisci from elderly dogs as previously described. Our goal was to evaluate the sensitivity of these MR parameters in detecting relatively mild degenerative tissue changes in mostly normally aged menisci and to determine whether and how these parameters can identify specific types of tissue alterations. If successful, these early markers could enhance the understanding of osteoarthritis pathogenesis and pave the way for more effective treatment strategies.

## 2 Materials and methods

### 2.1 Study samples

The stifle joints used in this study have been described previously (26). One joint had to be excluded from the original cohort of 16 joints from 8 dogs due to image artefacts. In total, 30 menisci from 15 joints (15 medial, 15 lateral) were included. Only elderly dogs, aged between 10 and 17 years, were selected for the study. None of the dogs had a history of hindlimb lameness or stifle instability. The reasons for the required euthanasia were unrelated to this study. Additional patient information can be found in Bunzendahl et al. (26).

Based on the results of X-ray scoring using the modified Kellgren-Lawrence scale (42, 43), 29% of the joints showed no radiographic evidence of osteoarthritis (score 0). The remaining 71% exhibited mild signs of osteoarthritis, corresponding to a score of 1.

Histological scoring was based on a system adapted from Sun et al. (44) and Pauli et al. (45), which includes individual scores for meniscus cellularity (0 to 3), collagen content (0 to 2), collagen organisation (0 to 3), and proteoglycan content (0 to 2). Additionally, a total score was calculated by summing up all the individual scores, resulting in a maximum possible total score of 10. Among the 30 menisci, one was classified with a total score of 0, while the highest total score observed was 6 (*n* = 2).

### 2.2 MR imaging

Before MRI, the stifle joints were dissected from the surrounding tissue and fixed in 10% neutral-buffered formaldehyde. The joints, submerged in the formaldehyde solution were positioned in a flexible 16-channel receive-coil (Variety, NORAS MRI products GmbH, Höchberg, Germany). All MR measurements were performed using a 3 Tesla whole-body MR system (MAGNETOM Prisma, Siemens Healthineers, Erlangen, Germany).

Maps of T2*-relaxation time were estimated from 3D multi-echo gradient echo (GRE) images acquired with the following parameters: TR = 75 ms, TE = 5–64.5 ms in 8.5 ms increments (8 echos), flip angle *α* = 25°, and an isotropic resolution of 300 μm. The total acquisition time was 1 h and 14 min. The T2*-maps were calculated using an in-house pixel-wise mono-exponential fitting routine (MATLAB R2021a, Math Works, Natick, MA, United States).

To estimate *MTR and MTsat*, 3D single-echo GRE images (TR = 27 ms, TE = 4 ms and 300 μm isotropic resolution) with varying weightings were acquired. Weightings were achieved by applying an off-resonance saturation pulse (MT-weighted: MT flip angle = 500° and MT offset = 1,500 Hz) or by modifying the flip angle *α* (proton density (PD)-weighted: α = 7°; T1-weighted: α = 20°). The total acquisition time was 27 min per acquisition. MTsat and T1 relaxation time were calculated as described in Helms et al. (37). Since the measured T1 relaxation time reflects both intrinsic tissue properties and the effects of the imaging technique, it is referred to as the *apparent T1 relaxation time (T1app).* T1app, T2*-relaxation time, MTR and MTsat were extracted from manually defined regions of interest (ROIs) that segmented the medial and lateral menisci. All segmentations were consistently performed by the same experienced observer on T2-weighted images using the software program ITK-SNAP.^1^ The segmentation process utilized sagittal, transverse, and coronal planes as previously described (26). An example of the segmentation is shown in Supplementary Figure 1.

### 2.3 Statistical analysis

Statistical analyses were conducted using the Python libraries statsmodels (version 0.14.1, www.statsmodels.org), scipy (version 1.10.1, www.scipy.org), and scikit-learn (version 1.3.0, http://scikit-learn.org). To assess differences in MR parameters across the three values (0, 1, 2) of each histological score, a one-way ANOVA was performed using statsmodels.stats.anova. Given the limited sample size and deviations from normality assumptions for some parameters (scipy.stats.shapiro), the ANOVA results should be interpreted with caution and are presented here primarily for orientation purposes. To ensure transparency, individual data points are displayed in the corresponding figure, enabling readers to assess the data distribution and variability within and across groups.

Additionally, a linear regression analysis was conducted using sklearn.linear_model, and the Pearson correlation coefficient was calculated with scipy.pearsonr. The normality of the residuals from the regression analysis was evaluated using scipy.stats.shapiro.

A paired *t*-test was applied to compare findings between the medial and lateral menisci using scipy.ttest_rel. A *p*-value of less than 0.05 was considered statistically significant.

## 3 Results

### 3.1 Delineation of menisci on differently weighted images and their derived maps

**Figure 1:**
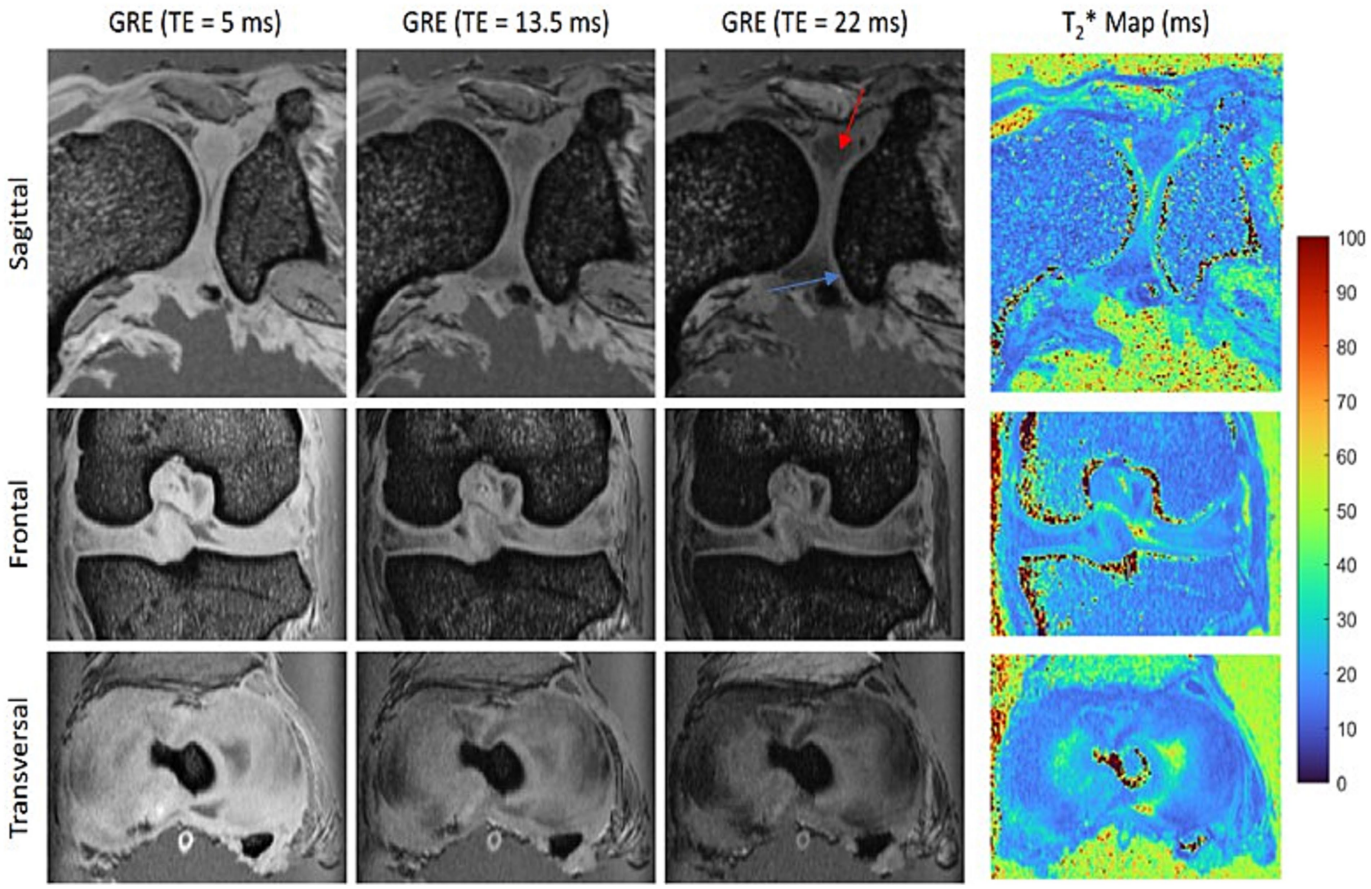
Orthogonal slices acquired with a 3D multi-gradient echo (GRE) sequence show the best contrast between the fibrous cartilage of the menisci (red arrow) and the hyaline articular cartilage (blue arrow) at an echo time (TE) of 22 ms. The corresponding T2* maps confirm the shorter T2* relaxation time of the menisci compared to the articular cartilage.

**Figure 2:**
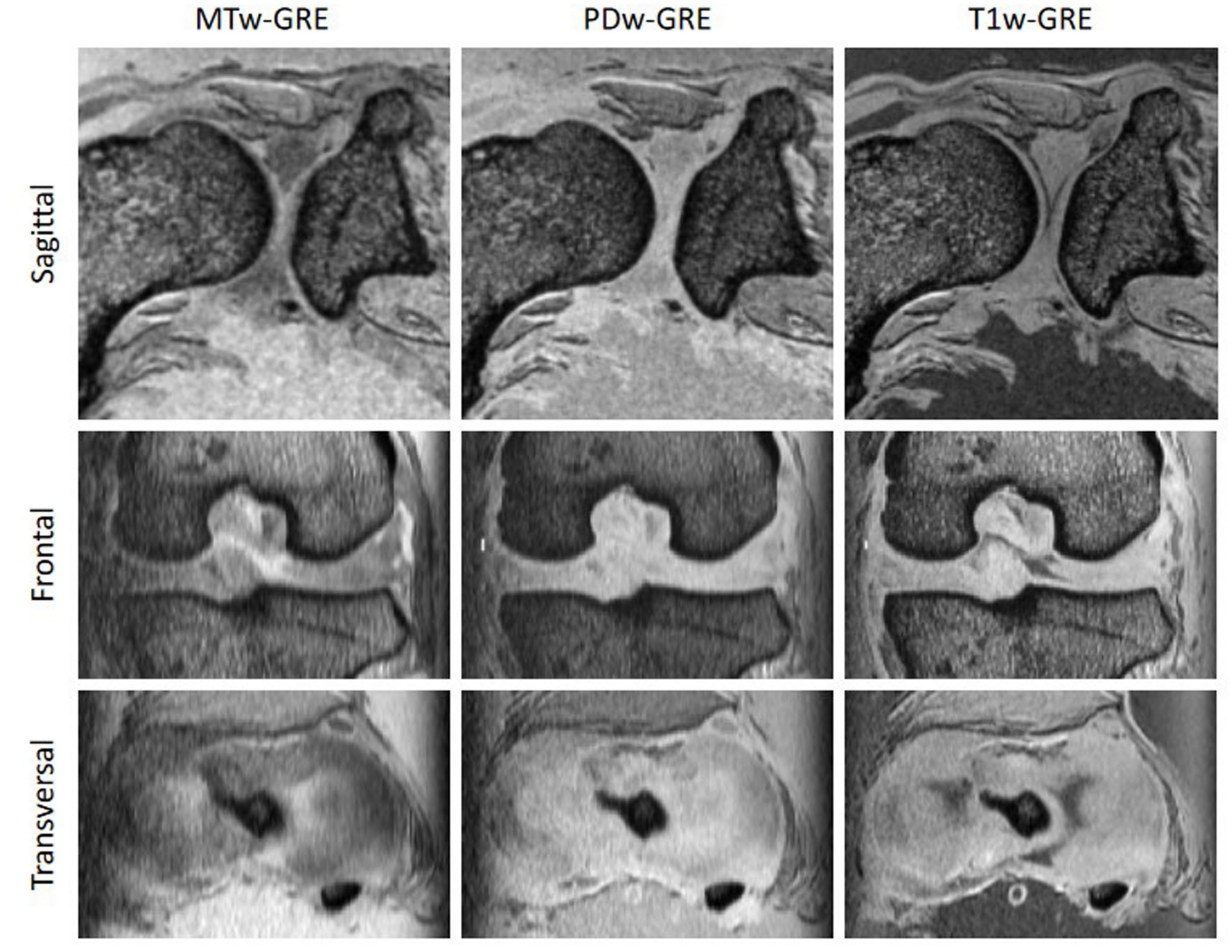
Comparison of images acquired with a 3D multi-gradient echo (GRE) proton-density weighted (PD) sequence with (MTw-GRE) and without (PDw-GRE) magnetisation transfer preparation, as well as the corresponding T1 weighted images (T1w-GRE). The macromolecule-based contrast of MTw-GRE provided the clearest delineation of fibrous and hyaline cartilage across all three orthogonal slices.

**Figure 3:**
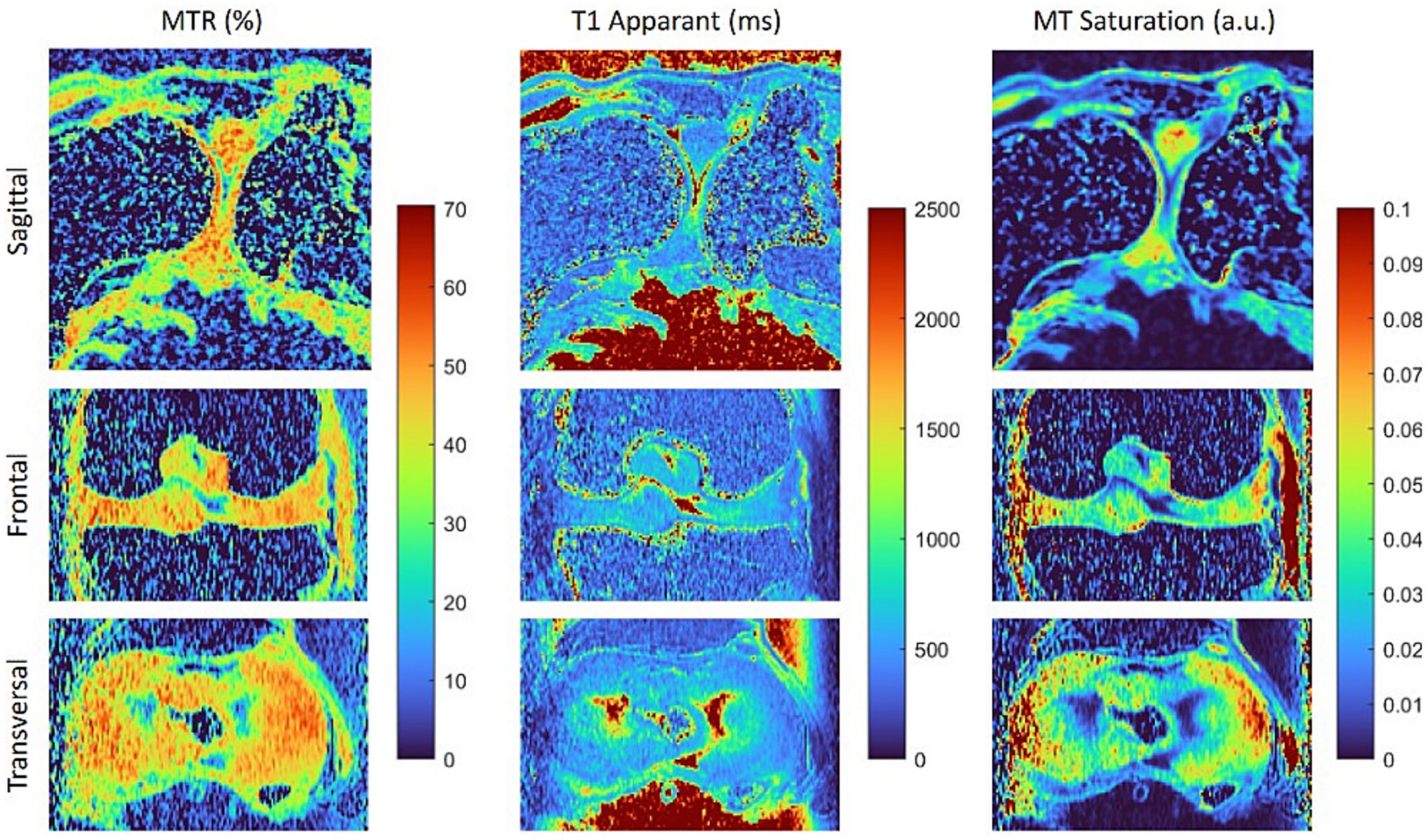
Maps of the magnetisation transfer ratio (MTR), apparent T1 relaxation time and magnetisation transfer saturation (MT saturation) calculated from the data sets shown in Figure 2. Both magnetisation transfer maps reveal differences between fibrous and hyaline cartilage, with the T1-corrected MTsat map offering enhanced contrast.

Figures 1–3 illustrate the achieved image quality, all derived from the same joint. The T2*-weighted images (Figure 1, GRE) clearly separate bone from softer tissues, including muscles and cartilage. As the echo time increases, the fibrous cartilage of the menisci (red arrow) becomes progressively better delineated from the hyaline articular cartilage (blue arrow), although the signal-to-noise ratio decreases. In the *post mortem* specimen, the best contrast was observed at TE = 22 ms. The improved delineation at longer TE is attributed to the shorter T2* relaxation time of fibrous cartilage compared to hyaline articular cartilage, as further confirmed by the calculated T2* relaxation time map (Figure 1, right).

Interestingly, the macromolecule-based contrast of magnetisation transfer-weighted imaging also clearly delineates fibrous and hyaline cartilage, whereas proton density-weighted (PDw-GRE) and T1-weighted (T1w-GRE) images show very little difference (Figure 2). The higher MT in fibrous cartilage is particularly evident on the calculated maps of MTR and MTsat (Figure 3). Notably, the T1-corrected MTsat map provides even better results than MTR, offering enhanced contrast and more precise tissue delineation.

### 3.2 Relationship between histological scores and MR parameters

**Figure 4:**
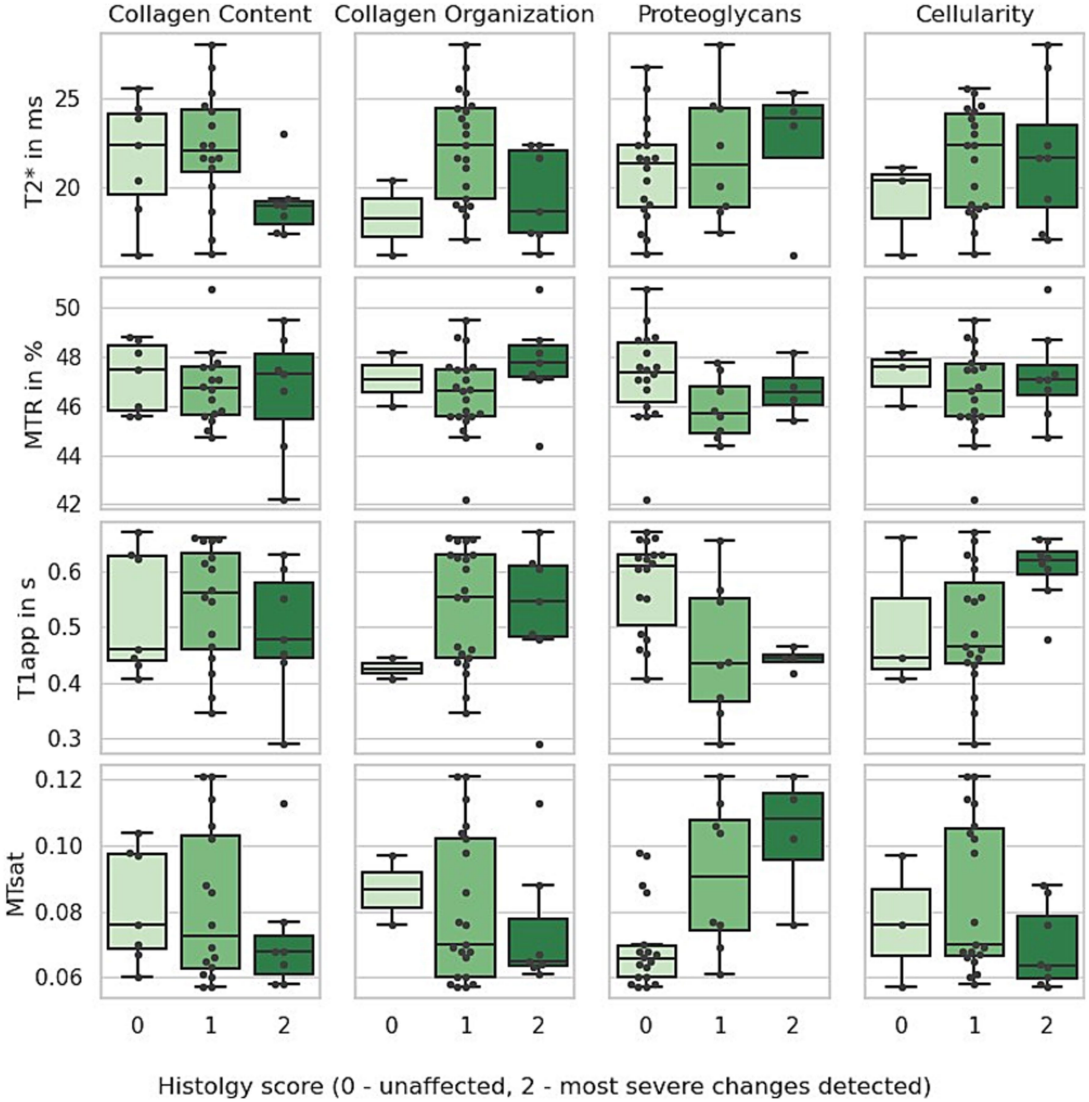
Boxplot of MRI parameters across different histological scores. The apparent T1 relaxation time (T1app) is shorter in menisci with increased proteoglycan staining and longer in those with increased cellularity score. In contrast, MTsat values (magnetisation transfer saturation) are higher in menisci with elevated proteoglycan scores.

The menisci included in this study exhibited histological scores for cellularity, proteoglycan content, collagen content, and collagen organisation ranging from 0 to 2. To assess whether these mild histological alterations were reflected in the MR parameters, the measured values were initially categorised according to the corresponding histological scores for each staining (Figure 4). A one-way ANOVA was performed primarily as an exploratory tool to provide an initial overview of potential differences in the MR parameters across the three scores. Additionally, the Pearson correlation coefficient was calculated to assess the relationship between each histological score and the corresponding MR parameter.

While most MR parameters did not show significant differences across individual scores, T1app decreased with increasing proteoglycan staining (ANOVA: *p* < 0.003; Pearson correlation: *r* = −0.54, *r* < 0.003) and increased with higher cellularity scores (ANOVA, *p* < 0.05, *r* = 0.37, *p* < 0.05). In contrast, MTsat values were higher in menisci with elevated proteoglycan scores (ANOVA, *p* < 0.0003, Pearson correlation: *r* = 0.63, *p* < 0.0003).

**Figure 5:**
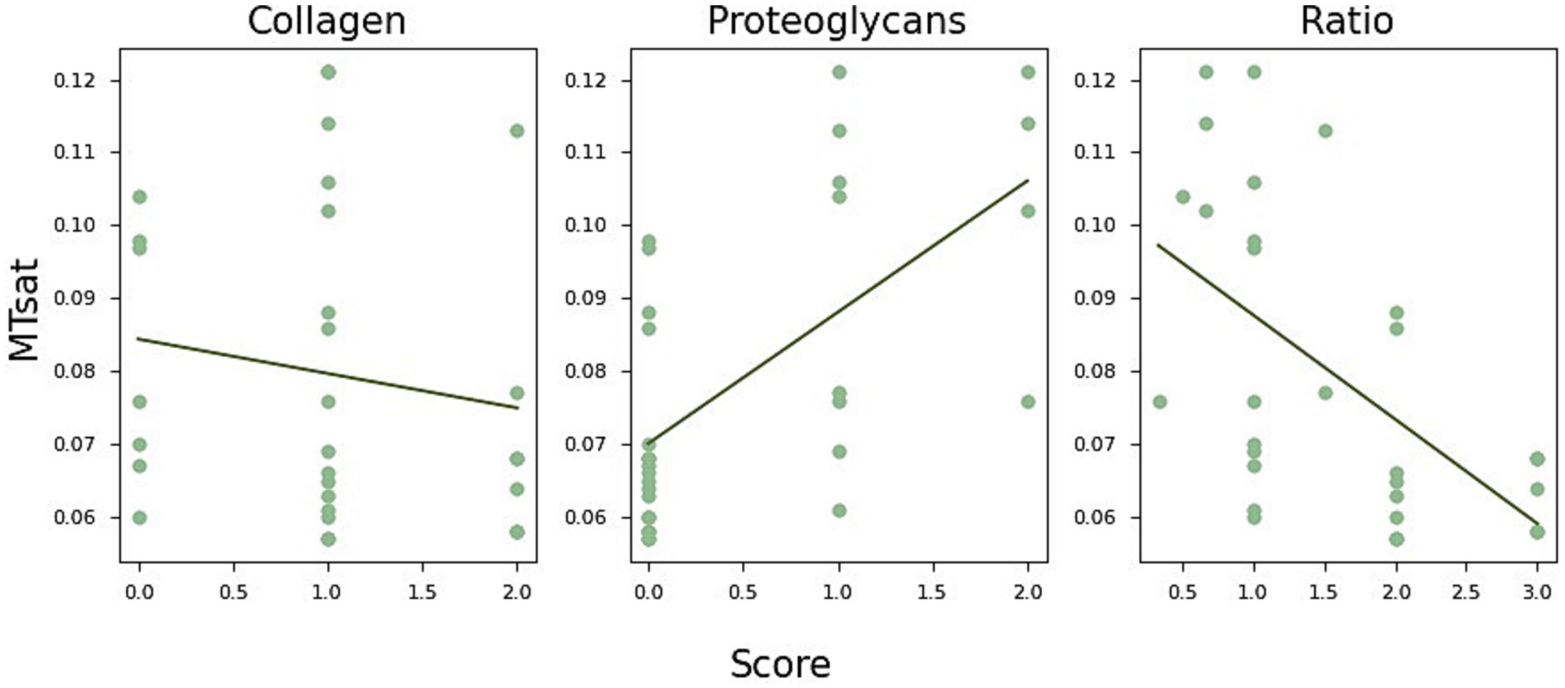
MTsat (magnetisation transfer saturation) values in relation to collagen and proteoglycan content. Collagen and proteoglycan scores appear to have opposite effects on MTsat. Specifically, MTsat tends to decrease with lower collagen content (*r* = −0.16, *p* = 0.43), while it increases with increased proteoglycan staining (*r* = 0.62, *p* < 0.0003). This relationship is further reflected in lower MTsat values associated with a reduced collagen-to-proteoglycan ratio (left, *r* = −0.56, *p* < 0.002).

Interestingly, collagen and proteoglycan content appeared to have opposite effects on MTsat. To explore this further, we calculated the ratio of the collagen and proteoglycan scores, adjusting all values by adding one to avoid division by zero. The results are shown in Figure 5, where MTsat significantly decreased with a higher collagen-to-proteoglycan ratio (*r* = −0.56, *p* < 0.002).

To investigate a potential correlation between the severity of changes and the MRI parameters, we summarised, as previously described (26), all individual histological scores into a total score and performed a correlation analysis between the total score and the MRI parameters. When analysing the lateral and medial menisci together, none of the parameters showed a significant correlation with the total score. A separate analysis of the lateral and medial menisci indicated a potential mild negative correlation between MTR and the total score in the medial menisci, though this trend did not reach statistical significance (*r* = −0.48, *p* = 0.08, Supplementary Figure 2).

### 3.3 Comparison of lateral and medial menisci

**Figure 6:**
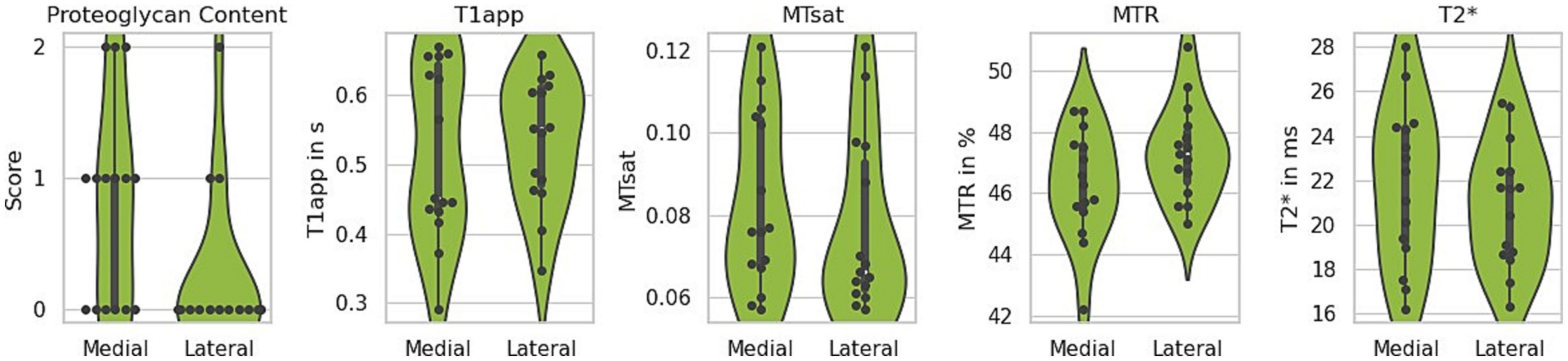
Comparison of lateral and medial menisci. The medial menisci exhibited more intense proteoglycan staining (higher score) and greater variability between the analysed samples. Among the quantitative MRI parameters, only the magnetisation transfer ratio (MTR) indicated a potential difference, showing a trend toward lower values in the medial menisci. T1app – apparent T1 relaxation time, MTsat – magnetisation transfer saturation.

Due to their restricted mobility, medial menisci are more prone to injury and may be more susceptible to degenerative changes over time (46). The histological analysis of the menisci included in this study revealed no statistically significant difference between the medial and lateral menisci (Supplementary Table 1), except for the proteoglycan score. On average, the medial menisci exhibited more intense proteoglycan staining (higher score), with greater variability between samples (paired *t*-test, *p* = 0.006) (Figure 6, left). To determine whether these histological differences are reflected in any of the MR parameters, the values for T2*, T1, MTR, and MTsat were analysed separately for lateral and medial menisci. None of the investigated parameters showed a significant difference, although MTR displayed a trend toward lower levels in the medial menisci (paired t-test, *p* = 0.059), as shown in Figure 6. The mean and standard deviation for each MR parameter, separated by lateral and medial menisci, are summarised in Table 1.

**Table 1:** Mean values and standard deviations (SD) of the respective magnetic resonance (MR) parameters (MTR – magnetisation transfer ratio, T1app – apparent T1 relaxation time, MTsat – magnetisation transfer saturation) in the medial and lateral menisci.

MR parameter	Medial menisci (mean ± SD)	Lateral menisci (mean ± SD)	*p*-value
MTR	46.3 ± 1.7 ms	47.4 ± 1.5 ms	0.06
T1app	517 ± 121 ms	536 ± 88 ms	0.56
MTsat	0.08 ± 0.02 ms	0.08 ± 0.02 ms	0.24
T2*	21.8 ± 3.4 ms	20.9 ± 0.7 ms	0.27

A paired *t*-test was applied to assess significant differences, and the corresponding *p*-values are provided.

## 4 Discussion

In this *ex vivo* study, we evaluated the detectability of mild, histologically confirmed degeneration in the menisci of elderly dogs with no history of hindlimb lameness using MRI. None of the included dogs showed signs of meniscal tears or other macroscopically visible injuries. Therefore, the samples can be considered representative of canine menisci from essentially normally aged dogs. Building on our previous study, which primarily investigated T2 relaxation times (26), we broadened our analysis to include T2* and T1 relaxation times, MTR, and MTsat. Our aim was to explore the suitability of these MR parameters for identifying mild changes in tissue microstructure associated with age-related degradation. Previous MRI studies on dog stifles have mainly concentrated on T2 and T2* weighted images or the corresponding relaxation time maps (1, 20, 21). To our knowledge, this is the first study on canine menisci that has additionally incorporated magnetisation transfer techniques.

Compared to hyaline cartilage, MRI of the menisci can be more challenging. Their lower water content, dense collagen network, and restricted proton mobility lead to rapid signal attenuation due to their short T2 and T2* relaxation times (29, 47). This also applies to *post mortem* imaging, as demonstrated in this study. Increasing the echo time of a gradient echo sequence, thereby enhancing T2* weighting, improved the delineation between hyaline cartilage and the menisci. However, excessively long echo times resulted in insufficient signal intensity. Furthermore, the MTR and MTsat values of the menisci were higher than those of hyaline cartilage, likely due to their dense collagen structure, offering a promising contrast mechanism for improved delineation.

The menisci analysed in this study exhibited relatively mild degenerative changes. The highest score observed in any histological staining did not exceed 2, and the highest total score was 6 (in 2 out of 30 menisci), with 10 being the maximum possible score. Our goal was to determine whether any of the tested MR parameters could reflect these subtle structural alterations. In summary, T2* relaxation times and MTR showed no significant differences across histological scores, while T1app and MTsat correlated with certain scores. These findings will be discussed in more detail below.

T2* relaxation time has been reported to increase following meniscal tears and other injuries. For instance, Koff et al. (48) observed prolonged T2* and T2 values in menisci after surgical repair in an ovine model. However, in the case of milder alterations, the results are less clear.

Nebelung et al. (29), for example, found no significant correlation between T2* values and histological degeneration in human menisci using the Williams grading system, which considers properties such as cellularity, matrix organisation and matrix staining intensity—a finding that aligns with our observations. A relatively mild increase in extracellular water, as opposed to the pronounced changes seen in acute injuries, combined with almost preserved fiber density and orientation, may render T2* less sensitive to detecting mild, age-related alterations. Furthermore, T2* relaxation is highly susceptible to magnetic field inhomogeneities. For example, differences in tissue fixation post mortem may overlay the effects of mild degeneration, contributing to increased standard deviation.

Interestingly, Pownder et al. (47) reported longer T2* values in the caudal horn of the medial, histologically normal menisci of healthy beagles. We did not observe any significant differences between lateral and medial menisci, although we did not analyse the horns separately. A possible reason for these differing observations could be the MR sequences used. The ultrashort echo time (UTE) imaging employed by Pownder et al. is more prone to the magic angle effect. This effect occurs when collagen fibres are orientated at approximately 55 degrees to the main magnetic field (B0), resulting in an artificially increased signal due to the decreased dipolar interaction of protons in collagen-rich tissues. The longer echo times used in our study may have minimised this effect.

Degeneration of the menisci is often accompanied by changes in collagen content and collagen organisation, making MT techniques a promising candidate for early detection. Zhang et al. reported significantly decreased MTR values in menisci from human patients with severe osteoarthritis. However, quantitative MT techniques appear to be more effective in detecting milder alterations (12). MTR can be affected by several confounding factors, including B1 inhomogeneity and T1 relaxation. It reflects both the exchange rate between free and bound protons and the recovery of longitudinal magnetisation of saturated spins. Consequently, when T1 relaxation times are shorter, the MT effect—and thus the apparent macromolecular content—may be underestimated, and vice versa (49).

The absence of significant changes in MTR in our study may be due to the relatively mild alterations in tissue properties. Additionally, the observed changes in T1 relaxation time could counterbalance the MT effect, potentially masking any detectable variations. Quantitative MT techniques aim to separate the effects of T1 relaxation from the MT effect, enabling parameters that more accurately reflect macromolecular content, such as the macromolecular fraction (MMF) or bound proton fraction (BPF).

Interestingly, Li et al. reported an increase in BPF associated with higher GAG levels in engineered cartilage (32), which aligns with our findings. The toluidine blue, which we used in this study to assess the amount of proteoglycans semi-quantitively, binds to the acidic GAGs within the proteoglycans. We found that menisci with increased proteoglycan staining showed higher MTsat values, a marker for magnetisation transfer that is corrected for T1 relaxation (37).

The role of GAGs in the pathobiology of meniscal tissue remains debated, especially in dogs, with limited data available. Inflammatory processes may increase enzymes like matrix metalloproteinases (MMPs) and aggrecanase, leading to a reduction in proteoglycan content. Conversely, there may be an initial rise in proteoglycan synthesis as the tissue attempts to repair, accompanied by the inhibition of degrading enzymes to support recovery (50, 51).

Notably, T1 relaxation time decreased with more intense proteoglycan staining and increased with higher cellularity scores, suggesting its potential for distinguishing between degenerative (reduced proteoglycan content, fibroblast-like cells) and regenerative processes (increased cellular activity and proteoglycan production) in the meniscus. Another promising diagnostic parameter may be the collagen-to-proteoglycan ratio, as a decrease in this ratio was linked to lower MTsat values. However, further studies involving menisci with a broader range of pathological lesions are needed to validate these findings. Future research should also compare MTsat and T1app directly with T1ρ in relation to the collagen-to-proteoglycan ratio, given T1ρ’s sensitivity to proteoglycan loss.

Besides the limitation of including only menisci with relatively mild changes and not having either healthy or severely affected menisci involved, the applied histological scoring system may also limit the correlation between MRI and histological findings. More quantitative, continuous readouts would be preferable. Further studies aim to address these limitations by including a broader range of pathological changes, a larger sample size, additional histological methods and separate analyses of the meniscal body and horns.

Another limitation of this study is that all measurements were conducted *post mortem* on formalin-fixed tissue. Formalin affects the tissue’s hydration state and thereby influences relaxation times (26, 29, 52). Additionally, measurements were performed at room temperature, which is lower than body temperature and impacts several MRI-relevant properties, such as exchange rate, diffusivity, and T1 relaxation time (53). On the other hand, *post mortem* MRI studies offer the advantage of longer measurement times, allowing for higher spatial resolution and the simultaneous use of multiple contrast mechanisms. So, this approach can be used to test a wide range of MR contrasts, with the most promising ones then applied and validated *in vivo*.

Finally, it should be noted that MR parameters are influenced by various factors, leading to limited overall specificity when detecting subtle or complex tissue changes. To achieve a more comprehensive characterization of meniscal degeneration—an essential factor in guiding therapeutic decisions—future studies should not only assess multiple MR parameters but also investigate their combined use. This integration could significantly enhance diagnostic specificity and precision. Based on our results, MTsat and T1app emerge as particularly promising parameters for future in vivo studies, which should encompass a broad spectrum of meniscal alterations, extending beyond age-related changes in canine menisci.

## 5 Conclusion

In summary, none of the investigated contrast mechanisms showed high sensitivity or specificity for detecting mild changes in the tissue microstructure of canine menisci, as assessed by histological analyses using the modified scoring systems of Pauli (45) and Sun (44). However, the observed correlation between MTsat and proteoglycan content may be a promising candidate for characterising the extracellular matrix, though further studies are needed to validate this effect.

## Data Availability

The raw data supporting the conclusions of this article will be made available by the authors, without undue reservation.

## Footnotes

^1^ www.itksnap.org
