# Supplementary material for: Magnetisation transfer, T1 and T2* relaxation in canine menisci of elderly dogs—an ex vivo study in stifle joints
Source: Front Vet Sci. 2025 Mar 12;12:1521684. doi: 10.3389/fvets.2025.1521684 (PMC11938368; doi:10.3389/fvets.2025.1521684)
Supplement: Supplementary file 1 [file Supplementary_file_1.docx]

# SUPPLEMENTARY MATERIAL

**Supplementary Table 1**: Mean values and standard deviations (SD) of the respective histology scores in the medial and lateral menisci. A paired t-test was applied to assess significant differences, and the corresponding p-values are provided.

| **Histology score** | **medial menisci  (mean ± SD)** | **lateral menisci  (mean ± SD)** | **p-value** |
| --- | --- | --- | --- |
| **Collagen content** | 1.1 ± 0.7 | 0.9 ± 0.7 | 0.43 |
| **Collagen organisation** | 1.1 ± 0.4 | 1.3 ± 0.6 | 0.19 |
| **Proteoglycan content** | 0.8 ± 0.7 | 0.3 ± 0.6 | **0.006** |
| **Collagen-to-proteoglycan ratio** | 1.4 ± 0.8 | 1.7 ± 0.8 | 0.1 |
| **Cellularity** | 1.1 ± 0.6 | 1.2 ± 0.5 | 0.72 |
| **Total score** | 4.1 ± 1.1 | 3.7 ± 1.5 | 0.31 |

**
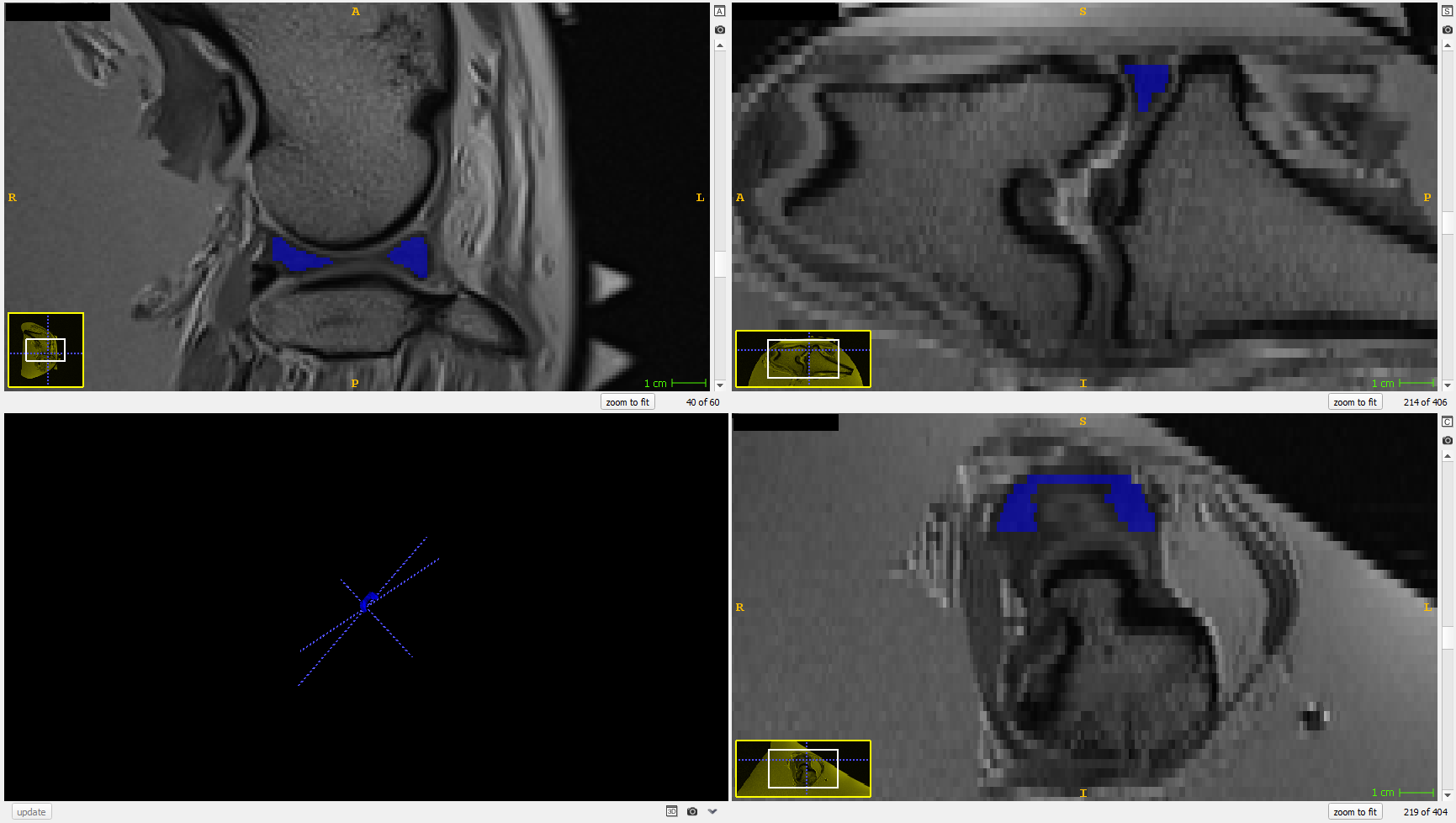
**

Supplementary Figure 1: Example of the manual segmentation of the lateral meniscus using the software program ITK-SNAP ([www.itksnap.org](http://www.itksnap.org)).

A
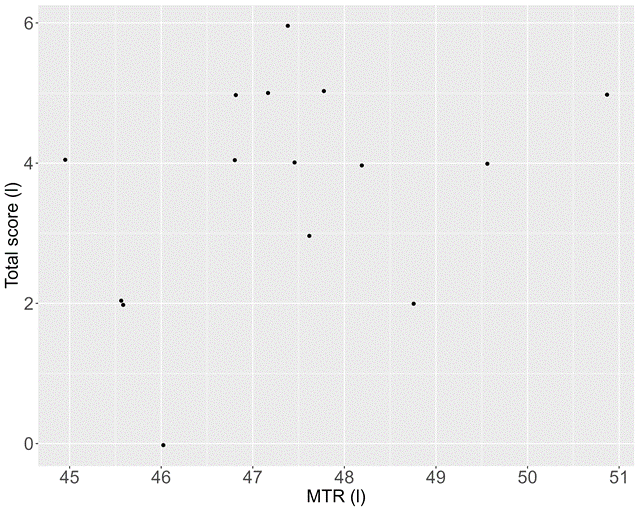
 B
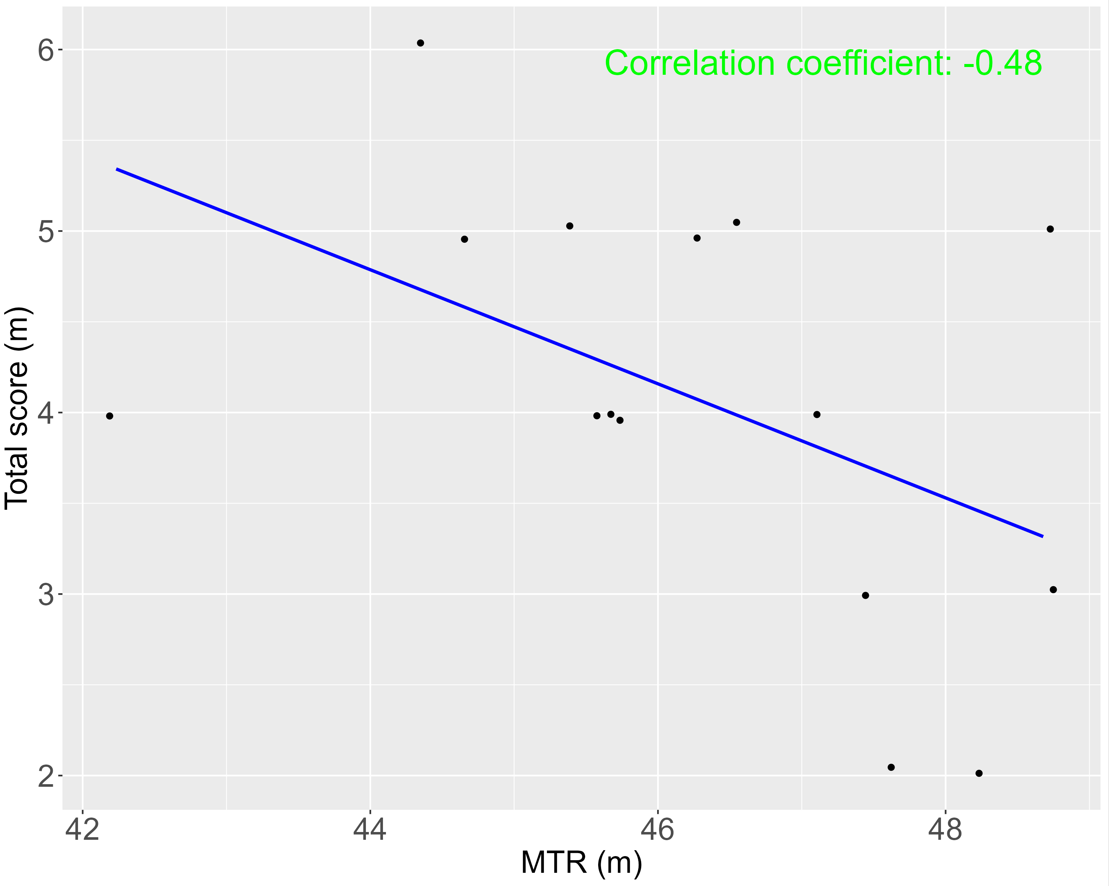


Supplementary Figure 2: Relationship of the total score (sum of all four single histological scores) and magnetisation transfer ratio (MTR) in (A) the lateral (l) and (B) medial (m) menisci.
